# Association between Childhood Maltreatment and Depressive and Anxiety Symptoms among Men Who Have Sex with Men in Los Angeles

**DOI:** 10.1007/s11524-023-00719-w

**Published:** 2023-02-24

**Authors:** David A. Wiss, Michael L. Prelip, Dawn M. Upchurch, Ondine S. von Ehrenstein, A. Janet Tomiyama, Pamina M. Gorbach, Steven J. Shoptaw

**Affiliations:** 1 Department of Community Health Sciences, Fielding School of Public Health, University of California Los Angeles, 650 Young Drive South, Los Angeles, CA 90095 USA; 2 Department of Epidemiology, Fielding School of Public Health, University of California, Los Angeles, 650 Young Drive South, Los Angeles, CA 90095 USA; 3 Department of Psychology, University of California Los Angeles, 502 Portola Plaza, Los Angeles, CA 90095 USA; 4 Department of Family Medicine, David Geffen School of Medicine, University of California Los Angeles, 10880 Wilshire Blvd, Los Angeles, CA 90024 USA

**Keywords:** Adverse childhood experiences, Childhood maltreatment, Childhood sexual abuse, Depression, Anxiety, Men who have sex with men

## Abstract

Adverse childhood experiences (ACEs) have been associated with poor mental health outcomes in adulthood. Childhood maltreatment is related to both depressive and anxiety symptoms. Our objective was to investigate these associations among low-income, mostly Black and Latino men who have sex with men (MSM), as these may be a particularly vulnerable population group. Data come from a longitudinal study of MSM with varied substance use behaviors (*n* = 321) collected between August 2014 and April 2022. Cumulative, childhood maltreatment ACEs, and the single ACE of childhood sexual abuse were investigated as potential predictors of self-reported depressive and anxiety symptoms in mixed-effects logistic and ordinal regression models. There was no evidence of a dose-response relationship between the number of ACEs and the predicted probability of depressive and anxiety symptoms. Compared to MSM reporting fewer than five ACEs, those with five or more ACEs had approximately double the odds ratio of reporting depressive (OR = 1.93; 95% CI: 1.04–3.60) and anxiety symptoms (OR = 2.21; 95% CI: 1.05–4.68). The dimension of childhood maltreatment had a more robust prediction of depressive symptoms than the dimension of household dysfunction across all models. The association between childhood sexual abuse history and depressive symptoms remained after adjustment for the other nine ACEs (OR = 2.27; 95% CI: 1.11–4.68). The ordinal logistic model suggested that cumulative ACEs more than triple the odds of being in a higher anxiety category (OR = 3.12; 95% CI: 1.58–6.14), with associations reported for childhood maltreatment ACEs (OR = 1.31; 95% CI: 1.06–1.66) and childhood sexual abuse (OR = 1.93; 95% CI: 0.89–4.21). Childhood maltreatment ACEs, particularly childhood sexual abuse, are salient predictors of depressive and anxiety symptoms among adult urban MSM. Mitigating the impact of childhood maltreatment requires understanding the additional burden of social distress often faced by MSM throughout the life course.

**Supplementary Information:**

The online version contains supplementary material available at 10.1007/s11524-023-00719-w.

## Introduction

Adverse childhood experiences (ACEs) recalled during the first 18 years of life have consistently been linked to poor mental health outcomes [1]. In North America, ACEs contribute to approximately 40% of depressive disorder cases and approximately 30% of anxiety disorder cases [2]. In a meta-analysis, the pooled odds ratio (OR) related to four or more ACEs for depressive symptoms was 4.40 (95% CI: 3.54–5.46) and for anxiety symptoms, 3.70 (95% CI: 2.62–5.22), compared to individuals with no ACEs [3]. Estimates from the most recent umbrella review suggest that the presence of any ACEs was associated with a two-fold increase in the odds ratio for depressive (pooled OR = 2.01; 95% CI: 1.86–2.32) and anxiety (pooled OR = 1.94; 95% CI: 1.82–2.22) symptoms [4].

While associations between total ACE scores and mental health outcomes have been well described, examining ACE categories using dimensional (subscales of ACEs) and selective (individual ACE) approaches may be informative to pinpoint targeted interventions. Evidence supports the use of ACE subscale scores to predict mental health disorders [5, 6]. In a longitudinal study, childhood maltreatment ACEs (emotional abuse, physical abuse, sexual abuse, emotional neglect, physical neglect) have been more closely associated with depressive and anxiety symptoms than household dysfunction ACEs (parental separation or divorce, mother treated violently, household substance abuse, household mental illness, and incarcerated household member) [5]. Among young adults from a nationally representative US longitudinal study, household dysfunction did not predict depression and anxiety disorders, whereas childhood maltreatment did [6]. In the recent umbrella review, the pooled OR for depressive disorders following exposure to any ACEs from the childhood maltreatment dimension was 2.02 (95% CI: 1.79–2.29), and for anxiety disorders 1.86 (95% CI: 1.62–2.14) [4]. From these findings, it appears that separating ACEs into dimensional subscales may elucidate links to depressive and anxiety symptoms better than using the sum score. The summed score has poor sensitivity to identifying synergistic interactions between adversities [7].

Individual ACE scores also correspond with mental health disorders in adulthood. Biopsychosocial pathways to poor health differ by the types of ACEs, with child physical, emotional, and sexual abuse often evincing the largest relationships [8]. Among children with documented physical and sexual abuse before the age of 12, earlier onset of maltreatment predicted more anxiety and depression symptoms in adulthood, adjusting for gender, race, current age, and other abuse reports [9]. Maltreated individuals typically develop psychiatric disorders at an earlier age, have more comorbidities and greater symptom severity, and respond less favorably to treatment efforts than non-maltreated individuals with the same diagnosis [10].

Meanwhile, ACE exposures are more likely to cluster with other forms of childhood adversity than occur in isolation [11]. For example, adults from racial/ethnic minority backgrounds and lower socioeconomic status (SES) frequently report high rates of ACEs [12, 13]. One study found that the prevalence of ACEs was higher among Black and Hispanic/Latinx youth than among White youths [14]. In this study, investigators added socioeconomic adversity to the traditional ACE measure to address the underestimation of the roles of childhood adversity on later-life mental health, particularly among racial/ethnic minorities. For example, racial discrimination perceived by Black children has been associated with diagnoses of depression (OR = 1.35; 95% CI: 1.23–1.49) and anxiety (OR = 1.39; 95% CI: 1.31–1.47) after adjusting for other ACEs and sociodemographic characteristics [15].

Associations between childhood maltreatment and adult depressive and anxiety disorders are greater for women than men, but differences from the meta-analysis are not statistically significant [16]. Meanwhile, studies investigating these associations among low-income Black and Latino men who have sex with men (MSM) are limited. Sexual minority groups are more likely to have experienced ACEs [17]. In one study, nearly 80% of MSM report exposure to at least one ACE [18], three times higher than the general population [2]. In another study, almost 90% of Black MSM experienced at least one ACE, and all ACEs were significantly associated with poor adult mental health outcomes [19]. More research on the impact of maltreatment among vulnerable populations is needed [20], particularly childhood sexual abuse among MSM.

Associations between urban MSM and depressive and anxiety symptoms are consistent in the literature, particularly among those living with HIV [21, 22]. It has been suggested that the diagnosis of HIV often precedes depressive symptoms [23]. Other determinants of depression among MSM living with HIV include HIV-related stigma, unemployment, current cigarette smoking, Black race/ethnicity, antiretroviral initiation, and access to mental health care [24].

Approximately one-third of MSM report elevated anxiety symptoms [25]. In the presence of ACEs, associations with anxiety and worse mental health quality of life have been reported among men living with HIV [26]. Links between ACEs and anxiety symptoms have been reported among urban MSM but not rural MSM [22]. There are fewer studies investigating associations between ACEs and anxiety symptoms among MSM than depressive symptoms. However, it has been established that anxiety disorders are associated with childhood sexual abuse among males [27], suggesting this ACE is noteworthy among MSM.

Among older adults at an outpatient HIV clinic, 40% reported experiencing sexual abuse in childhood [28]. MSM were nearly five times more likely to report childhood sexual abuse than male sexual nonminority individuals [29]. The prevalence of childhood sexual abuse is higher among MSM in racial/ethnic minorities [30, 31]. MSM with a history of childhood sexual abuse were more likely to be living with HIV and to report recent condom-less anal intercourse [32].

ACE scores have been associated with poor mental health outcomes, and marginalized groups such as MSM have been shown to be at higher risk for ACE exposure, exemplifying cumulative disadvantage over the life course. However, fewer reports investigate ACE dimensions separately and single ACEs (adjusted for the others) among MSM. The current cross-sectional study of low-income, mostly Black and Latino MSM first aimed to investigate whether the cumulative ACE scores were related to self-reported depressive and anxiety symptoms along a dose-response continuum. We further aimed to assess associations between the dimension of childhood maltreatment and household dysfunction, respectively, with depressive and anxiety symptoms and whether the single ACE of childhood sexual abuse was associated with depressive and anxiety symptoms.

## Methods

### Participants

The mSTUDY (Men Who Have Sex with Men and Substance Use Cohort at UCLA Linking Infections, Noting Effects (MASCULINE)) is an ongoing National Institute on Drug Abuse sponsored longitudinal study of HIV-positive and HIV-negative MSM with varied substance use behaviors (U01 DA036267). The mSTUDY was approved by the University of California Los Angeles Institutional Review Board. All individuals provided written informed consent at study entry. Because the current study is a secondary analysis from a previously approved study, this research was determined exempt. Prior work related to this project has been published [33].

Participants were eligible for enrollment if they were as follows: English-speaking, ages 18–45, assigned male sex at birth, if HIV-, reported having sex with men in the past twelve months, and enrolled at two community clinics in Los Angeles, CA. Staff at these sites determine if an individual qualifies and is interested in participating. Participants are compensated for study participation ($75 per study visit) and get access to free and confidential STI testing every 6 months, HIV risk-reduction counseling, testing, and referrals for care. By design, half of the sample is living with HIV, and the other half is HIV-. The mSTUDY investigates questions related to sexual health among Black and Latino MSM designed to ascertain information about HIV/STIs.

Study enrollment began in August 2014 and is ongoing. Participants complete assessments every 6 months, including a comprehensive physical exam and medical history, urine drug panel, clinical laboratory tests, and computer-assisted detailed behavioral questionnaire. ACE questions were added to the battery of behavioral data collected as part of the computer-assisted self-interviews on December 15, 2020, during remote visitation due to COVID-19 and 321 participants responded to the ACE questionnaire. The current study uses data on depressive and anxiety symptoms from the study conception (August 20, 2014) until March 13, 2020, when COVID-19 lockdowns began. Several covariates were not collected during remote visitation and were thus carried forward for analyses. While most covariates are likely stable over time (e.g., race/ethnicity, education level), others may change (i.e., HIV status, BMI), which is discussed as a limitation.

### Predictor

The ACE measure is a ten-item instrument [34]. Respondents answer yes/no to questions about childhood maltreatment (emotional abuse, physical abuse, sexual abuse, emotional neglect, physical neglect) and household dysfunction (parental separation or divorce, mother treated violently, household substance abuse, household mental illness, and incarcerated household member) experienced during the first 18 years of life. Subscales for childhood maltreatment and household dysfunction were analyzed as continuous variables (indexes ranging from 0–5). The single ACE of childhood sexual abuse was used for prediction in all models.

### Outcomes

#### Depressive Symptoms

The Center for Epidemiological Studies Depression (CESD) has 20 questions yielding a score from 0–60 [35]. The CESD cut-point for “likely depressed” <23/23+ is used to classify clinically meaningful symptoms linked with a likely diagnosis of depressive disorder [36]. CESD scores were collected since the study conception.

#### Anxiety Symptoms

Anxiety symptoms are operationalized using the GAD-7, a validated 7-item tool for screening for generalized anxiety disorder (GAD) and assessing its severity [37]. The sum score ranges from 0–21, with 0–4 indicating minimal anxiety, 5–9 mild anxiety, 10–14 moderate anxiety, and 15–21 severe anxiety. These categories are used for ordinal logistic regression, as well as the dichotomized measure (for logistic regression), which classifies those with scores of 0–9 as low anxiety and 10–21 as high (sensitivity = 89% and specificity = 82% from the validation study of a primary care sample across twelve US states) [37]. The GAD-7 was added to the mSTUDY on June 22, 2018.

### Covariates

*Age* was categorized as 18–29 (reference group), 30–39, and 40–52 years. *Race/ethnicity* was categorized as Black, White, Other Race, Hispanic/Latino (reference group). Other race includes Asian, Asian Indian, American Indian or Alaskan Native, and Native Hawaiian Pacific Islander, which was collapsed due to the small sample size. *Education* reflects the total number of years spent in school categorized as: did not finish high school (0–11 years), high school (12 years), some college (13–15 years), college grad+ (16 years) (reference group). *Income* was categorized as: $0–19,999, $20,000–39,999, $40,000+ (reference group). *HIV status* was measured using seropositive status from laboratory blood tests. *BMI* was categorized using the standard US definition: underweight (below 18.5), normal weight (18.5–24.99), overweight (25–29.99), and obese (30+). Because of the small cell size (*n* = 4), the underweight was collapsed into the normal weight category (reference group). BMI is included as a covariate because it has been shown to vary by ACEs [38], depressive symptoms [39], and anxiety [40]. *Cigarette/vape use* indicates if the participant reports current use of cigarette or e-cig/vape (combined into a single variable). Nicotine use is included as a covariate because it has been shown to vary by ACEs [41], depressive symptoms [42], and anxiety [43]. *Alcohol use* reflects frequency: in the past 6 months, how often did you have a drink containing alcohol? Answers include never, monthly or less, 2–4 times per month, 2–3 times per week, 4 or more times per week. Never and monthly or less were collapsed (reference group). *Drug test* urine drug screens (Fastect® II Drug Screen Dipstick Test D, Brenan Medical Corporation, Irvine, CA) identified recent use of methamphetamine, opiates, cocaine, ecstasy, marijuana, amphetamines, and fentanyl. A single indicator variable (i.e., yes/no for any positive on any drug test) was used for these analyses.

### Statistical Analysis

Sample characteristics were ascertained at the first visit, where ACE scores were collected (i.e., index visit). Several covariates were not collected at the same visit as ACE scores (i.e., age, race/ethnicity, education, HIV, and BMI). Multilevel commands using participant ID were used for mixed effects in random intercept logistic and ordinal regression models, where time (level one) is nested in person (level two). We defined statistical significance as *⍺* = 0.05, and all confidence intervals are reported at 95%. All analyses were conducted using Stata version 17 [44].

The first hypothesis of dose-response was examined using the continuous ACE score (categorical variable at each level of ACE) and the dichotomized CESD (<23/23+) and GAD-7 (<10/10+) in mixed-effects logistic regression models, adjusting for covariates. Stata’s post-estimation commands *margins* and *marginsplot* were used to retrieve and visualize predicted probabilities to assess a potential dose-response relationship. Once the best cut-point was determined from the margins plot (<5/5+), the full mixed-effects logistic models, including the entire set of covariates, were computed. For the outcome of anxiety symptoms, an ordinal logistic regression model was also estimated based on the proportional odds assumption (Supp. A).

The second hypothesis was examined using a dimensional approach, where ACEs were combined into indexes for childhood maltreatment and household dysfunction. First, childhood maltreatment was added by itself, then both ACE clusters were added to the logistic and ordinal regression models. To estimate differences between ACE dimensions, Stata’s post-estimation command *lincom* (using subtraction) was used to estimate the difference between estimates.

The third hypothesis was examined using a selective approach, with the single ACE of childhood sexual abuse used as a predictor variable in all models, as well as the other nine ACEs (indexed as a continuous variable) in the fully adjusted models.

## Results

Participant characteristics are summarized in Table 1 for the entire sample as well as by those who are likely depressed and those who report high anxiety. Those with the lowest income, currently using cig/vape, and tested positive on the drug test were more likely to be depressed and anxious.

**Table 1:** Characteristics of mSTUDY participants based on adverse childhood experience questionnaire responses between December 15, 2020, and April 18, 2022 (*n* = 321) by depressive and anxiety symptoms reported between August 20, 2014, and March 13, 2020

Characteristic	***n*** (%)	Likely depressed ***n*** (%^a^)	High anxiety ***n*** (%^b^)
Age			
18-29 years	59 (18.4)	15 (25.4)	12 (20.3)
30-39 years	154 (48.0)	45 (29.2)	31 (20.1)
40-52 years	107 (33.3)	33 (30.8)	26 (24.3)
Missing	1 (0.3)	0 (0.0)	0 (0.0)
Race/ethnicity			
Black	133 (41.4)	32 (24.1)	22 (16.5)
Hispanic/Latino	130 (40.5)	42 (32.3)	31 (23.9)
Other race	18 (5.6)	5 (27.8)	7 (38.9)
White	40 (12.5)	14 (35.0)	9 (22.5)
Education			
Did not finish high school	32 (10.0)	11 (34.4)	7 (21.9)
High school	108 (33.6)	29 (26.9)	26 (24.1)
Some college	94 (29.3)	29 (30.9)	19 (20.2)
College grad+	87 (27.1)	24 (27.6)	17 (19.5)
Income		*	*
$0–$19,999	160 (49.8)	61 (38.1)	45 (28.1)
$20,000–$39,9999	84 (26.2)	16 (19.1)	10 (11.9)
$40,000+	77 (24.0)	16 (20.8)	14 (18.2)
HIV status			
HIV-	135 (42.1)	32 (23.7)	23 (17.0)
HIV+	185 (57.6)	61 (33.0)	46 (24.9)
Missing	1 (0.3)	0 (0.0)	0 (0.0)
BMI			
Under or normal weight (< 25)	110 (34.3)	34 (30.9)	29 (26.4)
Overweight (25–29.9)	107 (33.3)	31 (29.0)	19 (17.8)
Obese (30+)	101 (31.5)	28 (27.7)	21 (20.8)
Missing	3 (0.9)	0 (0.0)	0 (0.0)
Cigarette/vape use		*	*
Not currently	224 (69.8)	52 (23.2)	37 (16.5)
Currently	97 (30.2)	41 (42.3)	32 (33.0)
Alcohol frequency			
Monthly or less (including never)	218 (67.9)	64 (29.3)	50 (22.9)
2–4 times/month	42 (13.1)	13 (31.0)	7 (16.7)
2–3 times/week	36 (11.2)	10 (27.8)	6 (16.7)
4+ times/week	25 (7.8)	6 (24.0)	6 (24.0)
Drug test^c^		*	*
Negative	(49.4)	(24.7)	(5.2)
Positive	(50.6)	(40.4)	(12.5)

^a^Relative to not likely depressed

^b^Relative to low anxiety

^c^Reporting % because *n* > 321 (collected prior to adverse childhood experiences)

*Significant chi-squared test at *p* < 0.05

The mean ACE score was 3.0 (SD = 2.8), and 72% of participants reported at least one ACE; 30.8% reported five or more. The most common ACEs were parents separated or divorced (50.5%), followed by emotional abuse (40.5%), household substance use (36.5%), physical abuse (34.9%), emotional neglect (30.8%), and sexual abuse (29.3%).

The mean CESD score was 17.9 (SD = 11.9), and 32.7% of participants were considered likely depressed during the reporting periods. Being likely depressed differed by income (with the lowest category of income having the highest likelihood of depressed), use of cigarettes or vapes (with those currently reporting use having a higher likelihood of depressed), and recent drug use (with those screening positive having a higher likelihood of depressed).

The mean GAD-7 score was 5.7 (SD = 5.8), and 23.1% of participants reported high anxiety symptoms (moderate or severe), while 8.9% reported severe anxiety in the 2 weeks before the assessment. Participants reporting high anxiety symptoms differed by income (with the lowest category of income having the highest likelihood of anxiety), cigarette/vape use (with those currently reporting use having a higher likelihood of anxiety), and drug screen (with those screening positive having a higher likelihood of anxiety).

### Dose-Response Association

**Fig. 1:**
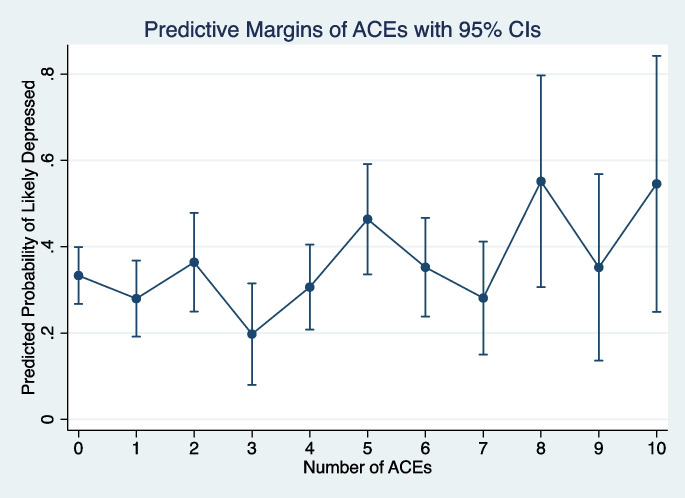
Margins plot from fully adjusted mixed-effects logistic regression of adverse childhood experiences on depressive symptoms reported between August 20, 2014, and March 13, 2020 (*n* = 318 across 2017 person-visits)

**Fig. 2:**
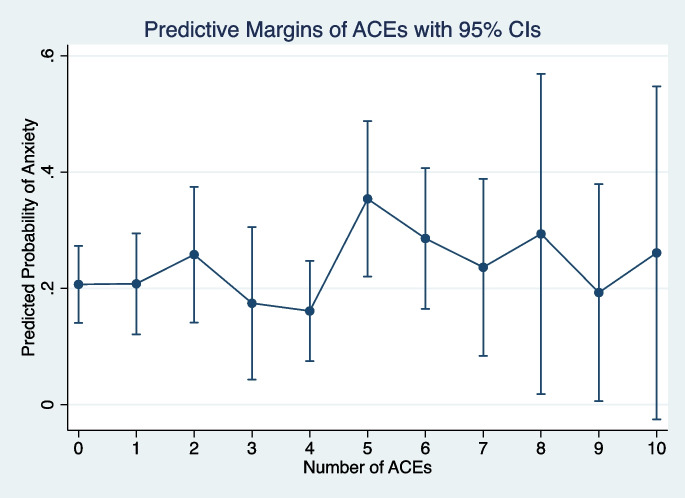
Margins plot from fully adjusted mixed-effects logistic regression of adverse childhood experiences on anxiety symptoms reported between August 20, 2014, and March 13, 2020 (*n* = 302 across 840 person-visits)

There was no indication of a dose-response association between the number of ACEs and the predicted probability of being likely depressed (Fig. 1) or having a higher level of anxiety symptoms (Fig. 2). However, these analyses determined that five was the optimal cut-point for models using total ACE scores (the point where the predicted probability of both outcomes tended to increase). The dichotomized ACE indicator (<5/5+) was then used to investigate whether the cumulative ACE score was associated with depressive and anxiety symptoms.

### Cumulative ACEs

#### Depressive Symptoms

Reporting of five or more ACEs (compared to four or fewer) was related to a greater odds ratio for being likely depressed (OR = 1.93; 95% CI: 1.04–3.60) after adjusting for age, race/ethnicity, education, income, HIV status, BMI, current cig/vape use, alcohol frequency, and drug use (Table 2); being exposed to five or more ACEs during the first 18 years of life was related to a nearly two-fold greater odds ratio for being likely depressed in adulthood.

**Table 2:** Association between adverse childhood experiences with depressive and anxiety symptoms among mSTUDY participants between August 20, 2014, and March 13, 2020

	OR	95% CI	*p*-value
Depressive symptoms^a^			
5+ adverse childhood experiences	1.93	1.04–3.60	0.04*
Childhood maltreatment index	1.19	1.00–1.41	0.05*
Childhood maltreatment index + HD index	1.24	1.01–1.53	0.04*
Childhood sexual abuse	2.25	1.20–4.23	0.01*
Childhood sexual abuse + 9 ACE index	2.27	1.11–4.68	0.03*
Anxiety symptoms^a^			
5+ adverse childhood experiences	2.21	1.05–4.68	0.04*
Childhood maltreatment index	1.18	0.95–1.46	0.13
Childhood maltreatment index + HD index	1.22	0.95–1.58	0.12
Childhood sexual abuse	2.25	1.05–4.78	0.04*
Childhood sexual abuse + 9 ACE index	2.32	0.98–5.49	0.06
Anxiety symptoms^b^			
5+ adverse childhood experiences	3.12	1.58–6.14	0.00**
Childhood maltreatment index	1.31	1.08–1.59	0.01**
Childhood maltreatment index + HD index	1.33	1.06–1.66	0.02*
Childhood sexual abuse	2.41	1.20–4.82	0.01*
Childhood sexual abuse + 9 ACE index	1.93	0.89–4.21	0.10

^a^Logistic regression adjusted for age, race/ethnicity, education, income, HIV, BMI, cig/vape, alcohol, drug use

^b^Ordinal regression adjusted for age, race/ethnicity, education, income, HIV, BMI, cig/vape, alcohol, drug use

*Significant at *p* < 0.05; **p < 0.01

*OR* odds ratio, *CI* confidence interval, *HD* household dysfunction

#### Anxiety Symptoms

Reporting of five or more ACEs (compared to four or fewer) was related to a greater odds ratio (OR = 2.21; 95% CI: 1.05–4.68) for having higher anxiety after adjustment (Table 2). Findings suggest that exposure to five or more ACEs during the first 18 years of life more than doubled the odds of high anxiety in adulthood. In the ordinal logistic model, the presence of five or more ACEs was related to a more than three-fold greater odds ratio (OR = 3.12; 95% CI: 1.58–6.14) of being in a higher anxiety category after adjustment (Table 3). This association appeared worse for White adults (compared to Hispanic/Latino adults), those in the lowest income category, and those screening positive for drug use on the urine test.

**Table 3:** Mixed-effects ordinal logistic regression of five or more ACEs on anxiety symptoms among mSTUDY participants between August 20, 2014, and March 13, 2020 (*n* = 302 across 840 person-visits)

Anxiety symptoms	OR	95% CI	*p*-value
5 or more ACEs	3.12	1.58–6.14	0.00**
Age	-	-	0.39
18–29 years	-	-	-
30–39 years	0.83	0.42–1.63	0.58
40–52 years	0.56	0.24–1.31	0.18
Race/ethnicity	-	-	0.00**
Black	0.55	0.27–1.10	0.09
Hispanic/Latino	-	-	-
Other	1.48	0.32–6.76	0.62
White	3.31	1.23–8.91	0.02*
Education	-	-	0.83
Did not finish HS	0.85	0.31–2.28	0.74
HS	0.77	0.37–1.57	0.47
Some college	0.73	0.37–1.43	0.36
College grad+	-	-	-
Income	-	-	0.00**
$0–19,999	3.38	1.61–7.10	0.00**
$20,000–39,999	1.99	0.96–4.13	0.07
$40,000+	-	-	-
HIV+	2.02	1.00–4.07	0.05*
BMI	-	-	0.28
Under or normal weight	-	-	-
Overweight	1.3	0.67–2.53	0.43
Obese	0.73	0.34–1.55	0.41
Current cig/vape use	1.60	0.93–2.77	0.09
Alcohol frequency	-	-	0.50
Monthly or less	-	-	-
2–4 times/month	1.49	0.88–2.54	0.14
2–3 times/week	1.25	0.61–2.58	0.54
4+ times/week	1.40	0.56–3.46	0.47
Drug screen+	2.46	1.50–4.03	0.00**
/cut1	1.68	0.57–2.79	
/cut2	4.06	2.91–5.22	
/cut3	6.01	4.78–7.23	

*p < 0.05; **p < 0.01

*OR* odds ratio, *CI* confidence interval, *ACEs* Adverse Childhood Experiences, *HS* high school, *Cig* cigarette

BMI: Underweight (< 18.5); normal (18.5–24.99); overweight (25–29.99); Obese (30+)

### Childhood Maltreatment ACEs

#### Depressive Symptoms

When separating childhood maltreatment and household dysfunction dimensions into continuous indexes and adding both variables to the fully adjusted model, only childhood maltreatment was positively associated with depressive symptoms (OR = 1.24; 95% CI: 1.01–1.53). For each additional childhood maltreatment ACE reported, the odds ratio for being likely depressed increased by 24%, adjusting for household dysfunction and other covariates (Table 2). Post-estimation results indicate that the linear combination of the parameters (using subtraction) did not reach statistical significance.

#### Anxiety Symptoms

The childhood maltreatment index was not associated with higher anxiety in the mixed-effects logistic regression model. However, in the ordinal logistic model (Table 3), childhood maltreatment was positively associated with being in a higher anxiety category (OR = 1.33; 95% CI: 1.06–1.66). The ordinal logistic model better predicted anxiety symptoms because it captured the likelihood of being in a higher anxiety category rather than the cruder binary estimate. For each additional childhood maltreatment ACE reported, the odds ratio for being in a higher anxiety category increased by 33%, adjusting for household dysfunction and other covariates. However, post-estimation results indicate that the linear combination of the parameters (using subtraction) did not reach significance.

### Childhood Sexual Abuse

#### Depressive Symptoms

In the model not adjusted for an index of the other nine ACEs (Table 2), childhood sexual abuse increased the odds of being likely depressed (OR: 2.25; 95% CI: 1.20–4.23). In the fully adjusted model, including an index for the other nine ACEs, childhood sexual abuse remained associated with depressive symptoms after adjustment (OR: 2.27; 95% CI: 1.11–4.68).

#### Anxiety Symptoms

In the logistic model not adjusted for an index of the other nine ACEs (Table 2), childhood sexual abuse increased the odds of having higher anxiety (OR: 2.25; 95% CI: 1.05–4.78). In the fully adjusted model, including an index for the other nine ACEs, childhood sexual abuse did not survive adjustment. The ordinal model followed a similar pattern, with childhood sexual abuse increasing the odds of being in a higher anxiety category (OR = 2.41; 95% CI: 1.20–4.82); however, 95% CI went below one after adjustment for the other nine ACEs.

## Discussion

This research suggests ACEs are associated with mental health outcomes during adulthood among diverse MSM in Los Angeles, California. The prevalence of individual ACEs in the study sample was consistently higher than in nationally representative samples [45]. Specifically, physical abuse was two times more frequent, and sexual abuse was nearly three times more frequent than reported in the Behavioral Risk Factor Surveillance System.

In the first set of analyses, cumulative ACE scores do not predict depressive and anxiety symptoms in a dose-response fashion in this cohort of predominantly Black and Latino low-income MSM in Los Angeles, CA. Instead, it appeared to be the presence of multiple ACEs and their cumulative impact that had predictive power. The original ACE study demonstrated a strong dose-response relationship between ACE score and the probability of lifetime and recent depressive disorders [46]. Longitudinal results from China also demonstrate a significant dose-response relationship between ACEs and adult depression [47]. There is a paucity of literature documenting dose-response associations between ACEs and anxiety, and none of the studies have been specific to MSM.

Because the association between ACEs and depressive and anxiety symptoms did not follow a dose-response continuum, the chosen models compared those with five or more ACEs to those below, supporting the concept of cumulative impact. Cumulative ACEs (dichotomized into higher versus lower exposure categories) predicted depressive and anxiety symptoms among study participants. Estimated odds ratios between multiple ACEs and depressive and anxiety symptoms from the current study are approximately half of those reported in meta-analytic findings comparing those with 4 or more ACEs to those with none [3]. Meanwhile, estimates from the current study are consistent with other findings that suggest that the presence of any ACEs roughly doubles the odds of both depression and anxiety [4].

Most analyses use an indicator for anxiety (yes/no) rather than a four-level outcome in ordinal logistic regression, which may partially explain divergent estimates. Many studies also use a lower GAD-7 cut-point (<5/5+) to indicate the presence of any anxiety. Meanwhile, five or more ACEs increased the odds of frequent anxiety by four-fold among urban, minority young adults from Chicago [48], which is consistent (but higher) with the findings reported in Table 3. Given the high-risk nature of the mSTUDY cohort and the potential contribution of other factors to anxiety symptoms (e.g., excessive worrying), it may be worth exploring the operating characteristics of different cut-points for classifying clinically meaningful symptoms of anxiety in this group and other high-risk MSM from various racial/ethnic minority backgrounds.

MSTUDY participants likely experienced other ACEs, such as peer victimization and discrimination (based on race and sexual orientation) outside the home, which are not captured by the current ACE instrument, potentially downwardly biasing estimates (due to misclassification bias) between ACEs and mental health. For example, in an urban sample of Black children, nearly 20% reported only community-level ACEs [49]. Thus, future research among urban diverse MSM should include community-level ACEs to capture a broader range of childhood adversity exposures to further investigate dose-response associations. Another example of a missed ACE is housing instability [50], which may have provided predictions more consistent with representative samples if included.

It is also likely that other social factors (i.e., low income and drug use) contribute to poor mental health in the absence of ACE exposure. Half the mSTUDY cohort was intentionally enrolled as actively using substances. Unassessed factors such as adult food insecurity and housing instability are known contributors to poor mental health [51] in addition to HIV-positive status [21, 22]. HIV-positive status was associated with anxiety symptoms in the ordinal logistic model (Table 3) but did not appear to play a role in models of depression (data not shown).

Given the call for dimensional approaches, our analyses found that the ACE dimension of childhood maltreatment was estimated as an important predictor of depressive and anxiety symptoms, whereas household dysfunction was not, providing partial support for the second hypothesis. Childhood maltreatment was a statistically significant predictor of anxiety symptoms in the ordinal but not logistic models. The addition of the household dysfunction dimension augmented estimates of childhood maltreatment across all models, but the difference between these predictors did not reach statistical significance.

One reason that childhood maltreatment can be more deleterious to mental health is through processes of biological embedding [52–57] that can increase inflammatory processes over the life course and thus contribute to risk for poor adult physical and mental health [58]. Many childhood maltreatment ACEs are physical assaults on the body, possibly contributing to greater effects on biological systems. One possible reason that household dysfunction did not emerge as predictive of depressive and anxiety symptoms may be because these ACEs generally correlate with low SES [59]. However, post hoc analysis removing income and education from the models did not affect estimates.

Given the interest in estimating the association of individual ACEs among specific subgroups, the single ACE of childhood sexual abuse (part of the childhood maltreatment dimension) emerged as a significant predictor of depressive symptoms in adulthood in the fully adjusted model, including the other nine ACEs, but it did not predict anxiety after adjustment for the other nine ACEs. Findings suggest that childhood sexual abuse had a better prediction of depressive rather than anxiety symptoms among diverse MSM. This finding has noteworthy implications for the impact of childhood sexual abuse on mental health (specifically depressive symptoms) among mSTUDY participants, which may warrant targeted interventions and suggest a need for expanded ACE screening among diverse MSM.

Childhood sexual abuse has received a disproportionate amount of attention in research compared to other ACEs. While some representative samples suggest that only 1.8–2.3% of men report childhood sexual abuse [60], other estimates suggest the prevalence is as high as 6.9% [61]. Adult males are less likely than adult females to report childhood sexual abuse [62, 63]. Stigma associated with homosexuality [64] as well as unhelpful disclosure responses [65] likely relate to more mental distress and may impair future disclosures. Among those reporting ACEs in the mSTUDY cohort, 28.7% experienced childhood sexual abuse. A higher prevalence of reported childhood sexual abuse among MSM in the mSTUDY suggests that this ACE may be particularly salient among this group and is likely not underestimated.

### Public Health Implications

Childhood adversity is a leading contributor to morbidity and mortality in the USA and may be considered a preventable determinant of decreased quality of life. One avenue through which ACEs can lead to poor health outcomes is compromised mental health status, which is known to amplify over the life course and generate inequities that widen health gaps between advantaged and disadvantaged groups. Recent estimates suggest that a 10–25% reduction in childhood maltreatment incidence could potentially prevent 30–80 million cases of depression and anxiety worldwide [4]. Minority groups such as urban MSM often face an additional burden of distress with multiple marginalized identities. Given that positive childhood experiences can buffer the impact of ACEs on mental health [66], community-based public health efforts should identify avenues to promote these positive experiences and prioritize groups such as low-income MSM at risk for HIV infection.

### Strengths

While the breadth of ACE research is vast among nationally representative samples, gaps remain among specific subgroups using dimensional approaches. This study contributes to the literature on diverse MSM with multiple forms of disadvantage. Including a wide range of sociodemographic and behavioral covariates allowed for considering a range of potentially important confounding factors in estimating the association between ACE exposure and mental health among urban MSM. Validated instruments captured the various constructs, and there were virtually no missing data.

### Limitations

The current study's cross-sectional and self-reported nature limited the assessment of early life experience as we cannot exclude recall and reporting biases, potentially resulting in over or under-reporting of ACEs. While mixed-effects regression models accounted for repeated measures, capitalizing on the longitudinal study design was not possible since many variables were collected at different time points. While most covariates are likely stable over time (e.g., race/ethnicity, education level), others may change (i.e., HIV status, BMI), which we could not account for in our analyses.

Examination of ACEs as yes/no, whether separately or combined into a sum score, limits the ability to examine the frequency in which ACEs occur and the differential impact this additional context can have on subsequent mental health outcomes. While our analysis separated ACEs into separate dimensions, using a sum score does not provide potentially important information about the synergistic interactions between these adversities [7]. New theoretical approaches to measuring ACEs, including weighting ACEs with more known pathology (e.g., childhood maltreatment), considering their timing (including discontinuity of the exposure), and severity/chronicity (e.g., neglect as an event versus ongoing condition) might improve prediction estimation [67]. Furthermore, even when families provide safety at home, children can experience adversity in the community, which was not considered in the current study. This includes neighborhood or school violence, bullying, and denigration in many forms, resulting from prejudice and/or “othering,” as well as stress caused by continuous exposure to discrimination and marginalization based on race/ethnicity and/or sex/gender as well as sexual orientation [68]. Future ACE research on MSM should include these expanded ACEs.

## Conclusions

Childhood maltreatment ACEs, particularly childhood sexual abuse, can significantly predict depressive and anxiety symptoms among adult urban MSM. Intervention strategies are needed to improve the upstream social context surrounding ACEs and identify buffering factors that offset risk trajectories. Mitigating the impact of childhood maltreatment requires understanding the additional burden of social distress often faced by MSM throughout the life course. Efforts to bring trauma-informed treatment to MSM should consider the higher prevalence of childhood sexual abuse among this group and tailor interventions to be gender-specific and culturally sensitive, aimed at fostering resilience and improving the mental health quality of life.

## Supplementary information

ESM 1 (DOCX 53 kb)
