# Supplementary material for: Association between Childhood Maltreatment and Depressive and Anxiety Symptoms among Men Who Have Sex with Men in Los Angeles
Source: J Urban Health. 2023 Feb 24;100(2):327–40. doi: 10.1007/s11524-023-00719-w (PMC9951846; doi:10.1007/s11524-023-00719-w)
Supplement: Supplementary file 1 — (DOCX 53 kb) [file 11524_2023_719_MOESM1_ESM.docx]

**Supplement A: Proportional Odds Assumption for Mixed-Effects Ordinal Logistic Regression using Categorical GAD-7 Outcome**

Usage of ordinal logistic regression requires the proportional odds assumption [1]. Estimates are assumed to be proportional (i.e., equal) for each level of the outcome. The ordinal model can be conceptualized as a series of logistic regressions for binary dependent variables. With the 4-category GAD-7, the model combines the comparisons: minimal anxiety to the higher three levels; minimal and mild compared to moderate and severe; and minimal, mild, and moderate compared to severe; into a single estimate. While there are several methods to formally test the proportional odds assumption with single-level data, current methods using Stata do not permit a formal test of the proportional odds assumption using mixed-effects ordinal logistic regression. It is a drawback that an appropriate goodness of fit test is not available for a multilevel proportional odds assumption, but efforts are currently underway [2]. Stata technical support was contacted, but they did not yet have an accepted method. Thus, an attempt to assess the proportional odds assumption was conducted two ways:

1. A multinomial logistic regression model followed by a post-estimation Wald test comparing estimates from each level to one another. Since mixed-effects models in Stata do not permit multinomial models, the *xtset* command was used to declare data as time-series along patient ID and visit number, as has been done previously in mSTUDY research [3]. Wald tests compared the estimates from mild, moderate, and severe categories (versus minimal) in both unadjusted and adjusted models, and a non-significant chi-square result (p>0.05) was used to determine if the proportional odds assumption could be satisfied.
   1. A post-estimation Wald test compared these estimates from each level to one another and yielded a non-significant chi-square result (p=0.96 in the crude unadjusted model; p=0.34 in the fully adjusted model) and was used as support for the proportional odds assumption.
2. A mixed-effects linear regression model using the four category GAD-7 to assess the impact of cumulative ACEs (<5/5+) on increasing level of anxiety. A significant positive estimate was used to assess if cumulative ACEs linearly predict being in a higher anxiety category.
   1. A significant positive estimate (ß=0.31; p=0.00) determined that cumulative ACEs (<5/5+) linearly predict a higher anxiety category. Visual inspection of the distribution of GAD-7 categories (Figure 1) and the ordinal nature of these levels was used to corroborate the proportional odds assumption.

**Figure 1:** **General Anxiety Disorder-7 (GAD-7) Categories Between June 22, 2018, and March 13, 2020** **(n=390 Across 1,046 Person-Visits)**

**References**

1. Brant R. Assessing Proportionality in the Proportional Odds Model for Ordinal Logistic Regression. Biometrics. 1990;46(4):1171.

2. Epasinghe N, Sooriyarachchi R. A Goodness of fit test for the Multilevel Proportional Odds model. Commun Statistics - Simul Comput. 2016;46(7):00–00.

3. Wiss DA, Javanbakht M, Li MJ, Prelip M, Bolan R, Shoptaw S, et al. Food Insecurity Partially Mediates the Association Between Drug Use and Depressive Symptoms among Men who have Sex with Men in Los Angeles, California. Public Health Nutr. 2021;1–26.
